# Health equity in an unequal country: the use of medical services in Chile

**DOI:** 10.1186/1475-9276-11-81

**Published:** 2012-12-18

**Authors:** Guillermo Paraje, Felipe Vásquez

**Affiliations:** 1 Business School, Universidad Adolfo Ibáñez, Santiago, Chile; 2 School of Business and Economics, Universidad del Desarrollo, Concepción, Chile; 3 Department of Economics, Universidad de Concepción, Concepción, Chile

**Keywords:** Equity, Inequality, Healthcare use, Chile, Household surveys

## Abstract

**Introduction:**

A recent health reform was implemented in Chile (the AUGE reform) with the objective of reducing the socioeconomic gaps to access healthcare. This reform did not seek to eliminate the private insurance system, which coexists with the public one, but to ensure minimum conditions of access to the entire population, at a reasonable cost and with a quality guarantee, to cover an important group of health conditions. This paper’s main objective is to enquire what has happened with the use of several healthcare services after the reform was fully implemented.

**Methods:**

Concentration and Horizontal Inequity indices were estimated for the use of general practitioners, specialists, emergency room visits, laboratory and x-ray exams and hospitalization days. The change in such indices (pre and post-reform) was decomposed, following Zhong (2010). A “mean effect” (how these indices would change if the differential use in healthcare services were evenly distributed) and a “distribution effect” (how these indices would change with no change in average use) were obtained.

**Results:**

Changes in concentration indices were mainly due to mean effects for all cases, except for specialists (where “distribution effect” prevailed) and hospitalization days (where none of these effects prevailed over others). This implies that by providing more services across socioeconomic groups, less inequality in the use of services was achieved. On the other hand, changes in horizontal inequity indices were due to distribution effects in the case of GP, ER visits and hospitalization days; and due to mean effect in the case of x-rays. In the first three cases indices reduced their pro-poorness implying that after the reform relatively higher socioeconomic groups used these services more (in relation to their needs). In the case of x-rays, increased use was responsible for improving its horizontal inequity index.

**Conclusions:**

The increase in the average use of healthcare services after the AUGE reform has not always led to improved equity in the use of such services in most services. This indicates that there are still barriers to the equitable use of healthcare services (e.g. insufficient medical human resources, financial barriers, capacity constraints, etc.) that have remained after the reform.

## Introduction

Among the Latin American countries Chile stands out for both its accelerated growth through the ‘90s and the improvements in its social indicators over the past decades. The per capita income in Chile during the 80s was similar to that of China and Jordan today, whereas the current average income is equivalent to that of Poland or Croatia (based on GDP per capita in dollars, year 2000) [1]. In terms of the Human Development Index, Chile received 45th place in 2010, the highest in Latin America. The analysis of the evolution of this indicator shows that Chile incremented its position greatly due to the increase in life expectancy. This indicator went from 69.2 years in 1980 to 76.8 years in 2000, and 78.8 years today, equivalent to that of developed countries, such as Denmark, Portugal, USA, etc. However, after this spell of growth with improvements in social indicators, Chile still remains one of the most unequal countries in the world (as measured, for instance, by the Gini coefficient) [1].

**Figure 1:**
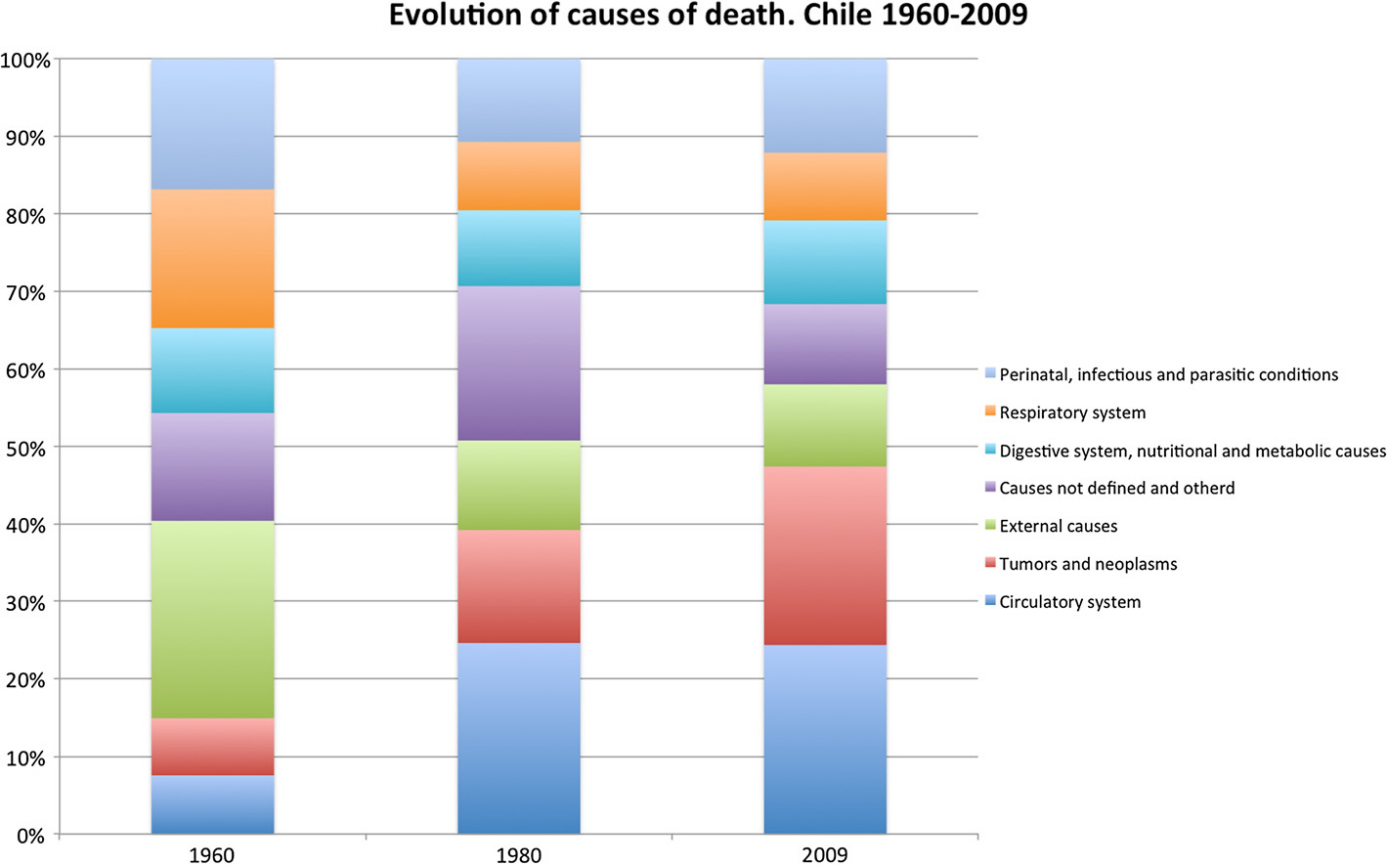
**Chile: Evolution of causes of death (1960–2009)[**[3]**].**

Health indicators also experienced favorable results and improvements. The infant mortality rate went from 23 in 1985 (similar to Nicaragua or Philippines today) to 7 in 2009 (similar to USA or Slovakia) [1]. Maternal mortality ratios also decreased from 56 in 1990 (equivalent to current levels in Brazil or Vietnam) to 26 in 2008 (similar to present day Turkey or USA) [2]. An accelerated demographic transformation was observed after improvements in health indicators, which ultimately changed the epidemiologic profile of the population. Figure 1 shows that the evolution of causes of death has varied between 1960 and 2009: cases linked to the elderly population, such as vascular problems or tumors have gained importance over those associated to respiratory, postnatal, or infectious diseases.

Over this period the Chilean health system underwent numerous reforms, yet only two of these merit special analysis. The first, implemented in 1979, created a private insurance system (Instituciones de Salud Previsional, also known as ISAPRES), which would coexist with a public insurance system, the only one present before the reform. The private system has a completely different logic compared to that of the public insurance system (of Bismarckian influence) because it started linking the individual insurance coverage with the payment capacity of each person. The private system began to select individuals based on their solvency and risk. On the other hand, the public insurance system (National Health Fund or FONASA) cannot take into account the financial solvency or payment capacity of its clients before determining the coverage given; it can only recollect a fixed percentage of the individuals’ income. This eventually resulted in a duality of insurance systems, in which the private sector gained relatively wealthier and healthier individuals while the public system kept the rest.

The second reform was implemented in 2005 and was aimed at correcting some of the defects presented in the Chilean health system, especially those that caused such unequal results. The objective of the AUGE reform (Spanish acronym for Universal Access with Explicit Guarantees) was to reduce existing socioeconomic gaps in the health system without greatly affecting the duality that has prevailed since the reform of '79. The AUGE reform did not seek to eliminate the private insurance system, but to ensure minimum conditions of access to the entire population for certain health conditions, at a reasonable cost and with a quality guarantee. In the context of a dual insurance system, universality of access would involve increasing fairness. After almost seven years since this reform was implemented there are virtually no studies that measure the impact of the AUGE reform on equity in the health system. There is no available data, at least to the public eye, on the subject and the few studies that have been published on this reform refer to the impact it has had on the aggregate use of medical services ([4,5]).

The aim of this paper is to provide an estimate on how equity in the use of healthcare services has changed before and after the AUGE Reform. This was done using household surveys conducted pre- and post-reform, which measure the use of certain health services (general practitioners, specialists, etc.). We estimated concentration and horizontal inequity indices for the use of each of these health services and decomposed the changes, following Zhong [6]. With the available data it cannot be argued that observed changes are entirely due to the impact of the AUGE reform and not to other concomitant factors. However, the article provides an exercise of comparative statics that can be seen as a first approach to the evaluation of the reform (if such evaluation is possible).

The article presents three contributions. First, the levels of inequality in terms of the use of health services were estimated for a set of variables pre-and post-reform. This makes the changes in relation to the use of such services for different socioeconomic groups evident. Second, the indices of inequality and horizontal inequity were estimated for an important range of variables of use, which have not been considered in previous studies in Chile. The fact that these variables were compared after a comprehensive reform of the health system adds a policy evaluation dimension that is new to Chile. Third, the indices of inequality and inequity were analytically broken down in order to identify the pure distributional impact of the observed changes. To the best of our knowledge none of these three issues have been previously addressed in the literature. The following section briefly describes the AUGE reform as well as evidence about its impact, in terms of use of medical care, improved chances of survival for different groups, etc. Section 3 presents the data sources as well as the methodology used in the analysis. Section 4 shows the results obtained followed by a discussion about their interpretations in Section 5. Finally, Section 6 presents the conclusions.

### The health system in Chile

**Figure 2:**
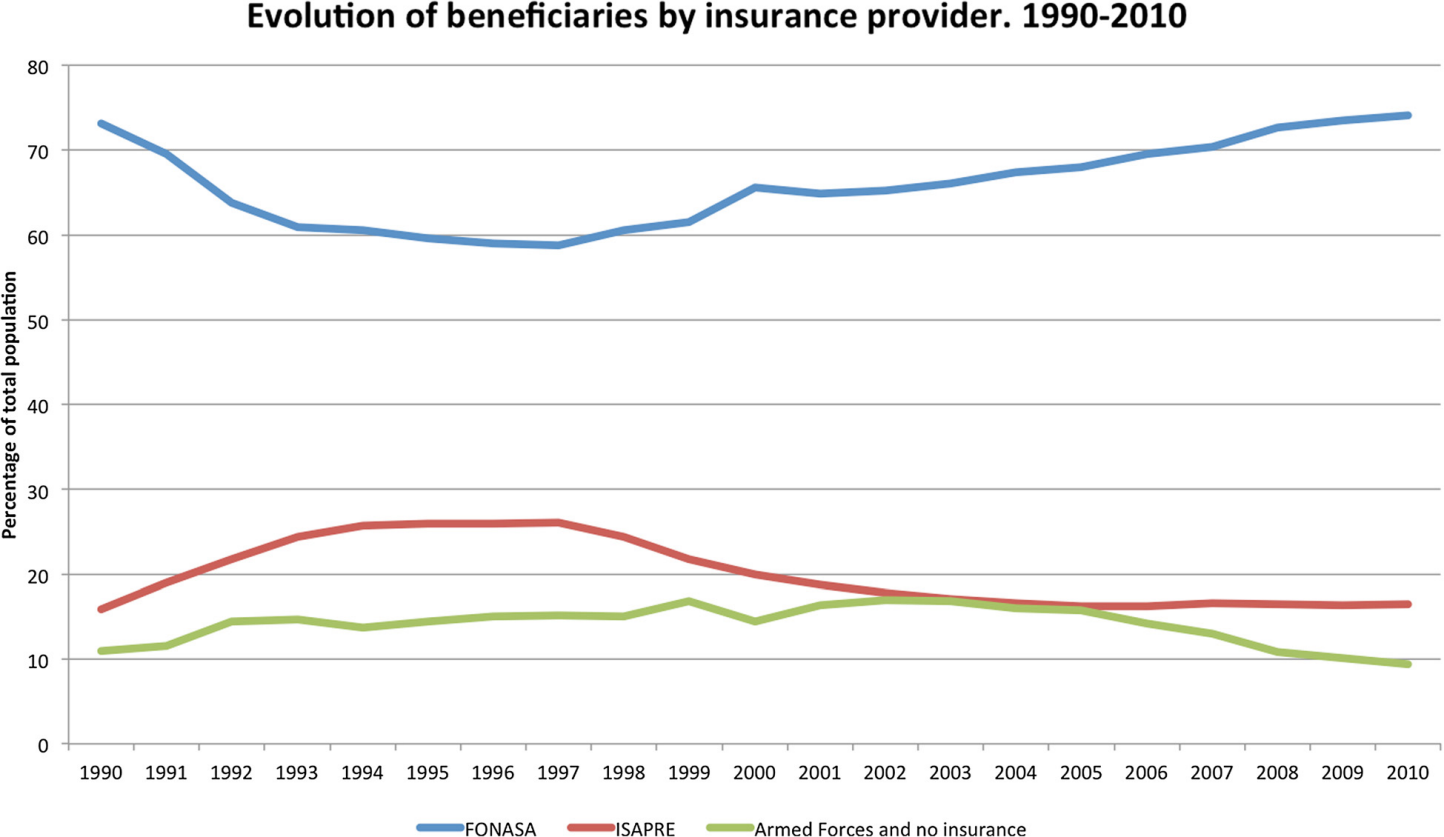
**Chile: Evolution of beneficiaries by insurance provider (1990–2010)[**[7]**].**

The Chilean health system is characterized by a dual system of insurance and healthcare benefits. The public insurance system, conducted by the National Health Fund (FONASA) is the most important in terms of beneficiaries. According to Figure 2, 74% of the population was insured by FONASA in 2010 after a strong increment starting in 1997. FONASA is financed by payroll contributions (7% of gross wages with a cap) and receives, in addition, a contribution from the Central Government (from general taxes) aimed to cover the insurance of people without payment capacity and the cost of having the fund to provide healthcare services. Within FONASA there are four types of insurances: the first two (Funds A and B) cover indigent and low income individuals (for these, public subsidies are complete or very high), while the remaining two (Funds C and D) are comprised of members who contribute (and for them the public subsidy is lower).

Private insurance is structured primarily around Provisional Health Institutions (ISAPRES) that in 2010 concentrated just under 17% of the population. As in the case of public insurance, ISAPRES receive 7% of the contribution of workers who choose to purchase such insurance and are accepted by these institutions. Affiliates of ISAPRES are free to contribute more than 7% of their gross wages if they choose to do so. In the case of ISAPRES, there is no public subsidy for those who wish to be a part of these institutions but do not have sufficient resources to afford the respective premiums.

An important part of the duality in the Chilean health system arises from the difference in the way both sub-systems function and the fact that there is no integration between them. Public insurance works as universal insurance would but without the “universality” simply because some people choose not to take part in it; however, it does have basic pillars of “universality” in terms of risk and income solidarity and the inability to deny insurance. The public subsidy also adds a moderately progressive redistributive element within it. On the other hand, private insurance runs on strict market logic: make sure the people that are insured can afford the premium and have a relatively low expected risk. The regulation of ISAPRES has been relatively lax, which has led to common practices of ex-ante (i.e. cream-skimming) and ex-post (e.g. risk adjustment for sex, age or health conditions) risk selection.

This coexistence of public and private insurance is found in many health systems, such as the UK, The Netherlands, etc. The difference between these and Chile is that in these countries there is no choice of leaving the universal insurance (usually public), which carries full risk solidarity. In these, private insurance is optional and complementary. In Chile, by contrast, private insurance is an alternative to the public one. This has at least two immediate effects; the first is that through the selection of risks ISAPRES have managed to keep an expected risk (cost) portfolio below the system average. Table 1 shows the population distribution among insurance systems according to autonomous income quintiles of the household, sex and age of its members.

**Table 1:** Population distribution by type of insurance, income quintile, gender and age groups

	**Household per capita income**					**Gender**		**Age**				
**Year 2000**	Quintile 1	Quintile 2	Quintile 3	Quintile 4	Quintile 5	Male	Female	0-17	18-44	45-64	65 and more	Total
FONASA	86.8	78.6	65.8	52.5	28.3	62.7	68.1	68.0	59.9	66.0	81.9	65.5
ISAPRE	3.7	8.9	18.4	30.7	55.8	21.5	20.1	21.8	23.4	19.2	6.5	20.8
Others/don’t know	9.5	12.4	15.8	16.8	15.9	15.8	11.7	10.2	16.7	14.8	11.6	13.7
TOTAL	100.0	100.0	100.0	100.0	100.0	100.0	100.0	100.0	100.0	100.0	100.0	100.0
**Year 2003**												
FONASA	90.9	85.2	75.9	60.0	33.2	69.2	74.0	74.9	66.8	71.4	83.5	71.7
ISAPRE	1.6	5.7	11.5	24.4	51.2	17.3	16.1	16.7	18.9	16.1	6.2	16.7
Others/don’t know	7.5	9.1	12.6	15.6	15.6	13.5	9.9	8.3	14.3	12.4	10.3	11.7
TOTAL	100.0	100.0	100.0	100.0	100.0	100.0	100.0	100.0	100.0	100.0	100.0	100.0
**Year 2006**												
FONASA	92.3	88.8	81.6	69.1	43.0	74.3	79.2	79.7	72.4	75.8	88.3	76.9
ISAPRE	2.2	4.0	8.0	17.0	44.1	14.3	12.7	13.4	15.8	13.2	4.3	13.5
Others/don’t know	5.4	7.2	10.4	14.0	12.8	11.3	8.1	6.9	11.8	10.9	7.4	9.7
TOTAL	100.0	100.0	100.0	100.0	100.0	100.0	100.0	100.0	100.0	100.0	100.0	100.0
**Year 2009**												
FONASA	93.2	90.3	85.1	72.3	44.6	76.6	80.8	81.5	75.0	77.3	87.5	78.8
ISAPRE	1.5	3.5	6.7	16.6	44.3	13.9	12.3	12.2	15.2	14.2	5.6	13.1
Others/don’t know	5.3	6.2	8.2	11.1	11.0	9.5	6.9	6.3	9.8	8.5	6.9	8.2
TOTAL	100.0	100.0	100.0	100.0	100.0	100.0	100.0	100.0	100.0	100.0	100.0	100.0

Source: Own calculation based on CASEN surveys.

From this table it is clear that between 2000 and 2009 ISAPRES concentrated individuals from the top two quintiles of income (because they can afford the premiums offered by ISAPRES), a proportion of men above the population average (due to lower relative risk of young men versus women of reproductive age) and youth (under 45 years, mainly). While 79% of the population was in FONASA (CASEN 2009) 87.5% of the population over 65 years belonged to the public insurance (only 5.6% were in ISAPRES). During the same year, while ISAPRES had 13% of the total portfolio, almost 45% of the portfolio belonged to the top quintile. Therefore, FONASA has concentrated those members who have higher relative risk/cost and lower incomes.

**Figure 3:**
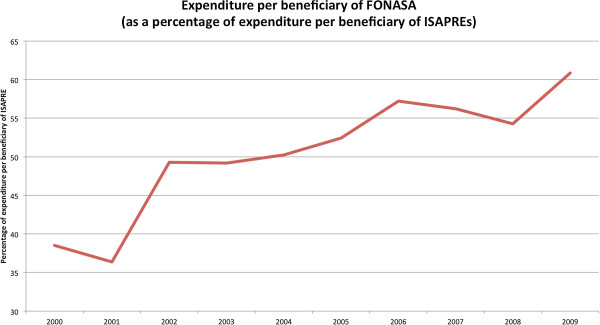
**Expenditure per beneficiary of FONASA (as a percentage of expenditure per beneficiary of ISAPREs)[**[7]**].**

The second effect is that the expenditure in health provisions per beneficiary (excluding out-of-pocket expenditure) is inferior for FONASA than for ISAPRES. The reason is simple: ISAPRES affiliates have greater relative ability to buy health insurance and have a lower risk/cost. Figure 3 shows the evolution of expenditure per beneficiary of FONASA as a proportion of expenditure per beneficiary of ISAPRES, which went from about 35% in 2001 to just over 60% by 2009. Despite the sharp increase in public spending in healthcare over the last decade, there is still a significant gap in the amount received by beneficiaries of both insurance systems.^a^

Finally, the provision of healthcare services also reflects a dual public and private partnership. The public system is structured under the National Health Service (SNSS), which besides the Ministry of Health includes 29 healthcare providers through a network of public hospitals and others by formal agreement. Primary healthcare is largely decentralized in the municipalities. Individuals without insurance can access the public healthcare network so, in practice, FONASA acts as a “last resort” insurer.

Furthermore, there is a network of for-profit private providers (independent professionals, clinics, hospitals, etc.). Most users of the private healthcare system are ISAPRES beneficiaries, beneficiaries of FONASA groups C or D, or individuals on waiting lists of guaranteed healthcare services.

Duality in insurance and provision did not impede the improvement of the population’s health indicators (thanks to good epidemiologic control), but it did generate great inequalities in the distribution of these health gains. Vega shows that towards 1998 when health indicators had already improved considerably, the infant mortality for children who had mothers with no formal education was almost 6 times greater than the mortality for those who had mothers with formal education [8]. The causes of death for the population generally associated with neoplasms or cardiac diseases showed an inverse correlation with the level of education. The National Health Survey in 2003 also revealed variations in the prevalence of certain risk factors depending on socioeconomic level. Hypertension, for example, was 70% more likely in lower socioeconomic groups; obesity was 60% more prevalent in such groups; and finally, diabetes was 200% more likely [9]. In part these differences can be explained by structural social determinants, but also because of differentiated access to the health system.

### The AUGE health reform

The gradual implementation of a health reform in Chile, known as AUGE, began in 2005. The aims of this reform were manifold: to reduce the accessibility gaps identified between different socioeconomic groups; to give greater priority to policies aimed at population-levels, emphasizing health prevention; to guarantee access to quality and timely care at a non-catastrophic cost for high-impact health problems [4]. The proposed reform sought to achieve these objectives without changing the fundamental organization of the health sector. There was a failed attempt to change the logic of this dual functioning insurance market (through the implementation of a Risk Compensation Fund between FONASA and ISAPRES that would have allowed the free movement of members between the different insurance companies), yet this initiative was politically boycotted [10].

The core of the AUGE reform consisted in defining a list of health conditions for which universal access (independent of insurance choice) and timely care was guaranteed, with a guaranteed quality (from the protocols defined for each condition) and with a guarantee of financial protection if the cost of the treatment exceeded a specified amount. The initial list included 25 conditions and the guarantee started in mid-2005. Fifteen more conditions were added in 2006, 15 more were added in mid-2007 and finally 10 more by mid-2010. Currently there are 66 guaranteed health conditions, with plans to expand the list to 80.

Guaranteed conditions were chosen on a multi-criterion basis, in which epidemiological criteria (those for which the years of potential life lost were greater), cost-effectiveness (diseases for which existing treatments were cost-effective), and sufficient coverage (possibility of covering potential demand at national level) were considered. Some diseases (like HIV/AIDS, for instance) were included not because they met the above criteria, but because of the social impact they have (social preference criterion). However, not all criteria were given the same weight. Vargas and Poblete show that among the first 56 conditions guaranteed almost 90% of them satisfied the epidemiological criteria and the social preference criterion with other criteria being far less important than these [11]. The prioritization meant that the first 56 conditions guaranteed represented 73% of the burden of disease, 51% of hospital discharges in the public system in 1996 and 60% of deaths in 1999 [5].

Since its implementation the guarantee system has served just over 11.8 million cases (until March 2011) of which almost 95% are FONASA beneficiaries; 81% of the cases treated were outpatients, 11% were hospitalizations and the rest are mixed cases. Five diseases account for 62% of total cases: hypertension (17%), acute respiratory infection (16%), outpatient dental emergency (16%), depression in people over 15 years old (7%) and diabetes mellitus type II (6%).^b^ Within these conditions the percentage of ISAPRES beneficiaries who took the guarantees varies considerably. Acute respiratory infection (ARI), for example, reaches only 3% of the total cases (both ISAPRES and FONASA beneficiaries) and 6% for hypertension and depression at age 15 and over accounts for only 14% of the total cases [12]. This suggests that ISAPRES users would have used guarantees (which means receiving attention in clinics or hospitals which were pre-established by insurers and providers rather than by beneficiary election) mainly as a complement to their individual insurance plans. In cases where individual insurance coverage is not important, as in the case of psychiatric care, users would have chosen AUGE coverage. For cases were private coverage was important, such as ARI, individuals chose to use private insurance instead of the AUGE modality (freely choosing their healthcare provider). Table 2, based on CASEN 2009, shows that for ISAPRES beneficiaries the main reason they have not taken part in AUGE is because they would rather personally select their specialist or facility (35%). The existence of prior coverage explained 8% of cases and has the highest percentage among ISAPRES affiliates. By contrast, among members of FONASA A and B the main reason was linked to lack of knowledge regarding the guarantees and what they included. Urrutia et al. studied this problem for the use of Pap tests, finding similar reasons for non-use of guarantees as those described in Table 2[13]. Missoni and Solimano pointed out that in several cases medical practitioners discouraged AUGE use, which is also shown in Table 2[14].

**Table 2:** Reasons for not using AUGE healthcare guarantees

	**FONASA A + B**	**FONASA C + D**	**ISAPRE**	**None**	**Another/Don’t know**
Preferred another practitioner/clinic	13.7	23.9	34.7	17.0	26.2
Chose not to wait	8.7	16.4	6.7	5.0	4.6
Thought AUGE would be of inferior quality	2.2	6.1	6.0	9.0	0.9
Previous insurance coverage	2.2	1.8	7.9	2.2	7.9
Paperwork to access to long	5.2	5.0	3.5	6.1	1.4
Not included in AUGE guarantees	3.2	3.2	4.1	5.3	1.5
Did not know about AUGE guarantees	37.8	19.4	16.8	17.8	15.6
Another age Group	2.6	3.3	1.0	0.8	2.2
Medical doctor did not recommended AUGE	3.7	1.3	0.9	0.0	1.7
Another reason	20.6	19.5	18.4	36.9	38.1
Total	100.0	100.0	100.0	100.0	100.0

Source: Own calculation based on CASEN 2009.

Table 3 shows how the number of consultations has evolved between 2006 and 2010 for the initial diseases and treatments included in AUGE. Acute Myocardial Infarction for example, has had an explosive increase (over 100% average annual growth in FONASA). Also, acute respiratory infections had an average annual growth of 28% in FONASA and 33% in ISAPRES. This does not imply an increase in the prevalence of these conditions at a general level and not necessarily an increase in the number of consultations, as most of them were previously attended in both the public and private system. What it probably does imply is that there is a reallocation of resources within both the public and private system as to attend to them within the guaranteed timeframe. In other cases, such as schizophrenia or cataracts, coverage levels particularly in the private system were relatively low and the introduction of AUGE led to an increase in accessibility for these conditions.

**Table 3:** Evolution of medical consultations for selected AUGE conditions

	**Cases July 2005/June 2006**		**Cases July 2006/June 2007**		**Cases July 2007/June 2008**		**Cases July 2008/June 2009**		**Cases July 2009/June 2010**		**Average Annual Growth 2005-2010**	
**Health condition**	**FONASA**	**ISAPRE**	**FONASA**	**ISAPRE**	**FONASA**	**ISAPRE**	**FONASA**	**ISAPRE**	**FONASA**	**ISAPRE**	**FONASA**	**ISAPRE**
Terminal Chronic renal Insufficiency	2,026	151	2,832	305	3,210	184	3,400	394	3,276	285	17.4	23.6
Congenital Cardiopathies	3,077	182	5,917	338	6,894	233	10,558	438	7,287	348	33.3	24.1
Cervical Uterine Cancer	8,737	569	14,122	938	15,039	1,088	14,460	1,540	15,429	1,441	20.9	36.3
Palliative care for terminal cancer patients	5,787	177	11,874	475	13,227	532	14,116	981	14,290	869	35.2	70.0
Acute myocardial infarction	10,286	421	43,218	736	74,129	509	82,039	1,279	84,664	957	101.9	31.5
Diabetes Mellytus type 1	717	467	701	491	775	787	788	766	810	521	4.1	3.7
Diabetes Mellytus type 2	85,022	7,634	91,496	9,900	80,156	7,416	72,601	9,113	70,357	7,280	−6.1	−1.6
Breast cancer	4,366	911	8,327	2,093	9,357	2,110	11,647	2,641	9886	2,234	31.3	34.9
Spinal Dysraphism	214	8	458	27	531	40	1,128	49	1,059	30	70.4	55.4
Scoliosis, Surgery in patients younger than 25	150	73	400	131	449	62	1,261	248	563	157	55.4	29.1
Cataracts	16,353	712	36,358	1,521	55,145	1,724	86,929	2,338	51,294	2,069	46.4	42.7
Severe Hip Arthritis requiring replacement	708	74	1,233	128	1,727	99	4,018	178	2,024	139	41.9	23.4
Cleft lip and palate	177	20	298	34	339	19	498	59	301	44	19.4	30.1
Children cancers	542	57	947	118	914	67	1,551	155	1,089	125	26.2	29.9
Schizophrenia	992	75	2,115	211	2,520	240	4,420	291	2,705	262	39.7	51.7
Testicular cancer	792	135	1,631	230	1,681	200	2,632	290	1,490	287	23.4	28.6
Lymphomas in adults	599	109	1,215	228	1,418	207	2,094	316	1,662	297	40.5	39.7
Acute respiratory infections in children	145,404	4,464	337,215	10,005	277,354	7,038	333,748	12,452	306,583	10,697	28.2	33.8
Community-acquired Pneumonia with outpatient treatment	10,487	39	25,522	100	19,768	60	22,609	105	20,721	55	25.5	12.1
Arterial Hypertension	262,695	14,667	246,300	22,170	216,376	15,249	185,628	24,621	169,272	17,246	−13.6	5.5
Nonrefractory Epilepsy	769	103	1,006	213	779	267	723	150	676	177	−4.2	19.8

Source: Own calculation based on FONASA statistics.

In terms of costs, it is estimated that by 2010 eight diseases concentrated half of the expenses for the entire system (public and private) [5].^c^ A significant proportion of these diseases are preventable, which means that by taking widespread measures, such as educational policies and prevention programs, these costs could be greatly reduced.

Once this reform was implemented significant waiting lists emerged for some conditions. For example, in July 2010 there was a waiting list of more than 183,000 patients (about 1.75% of the consultations for the guaranteed conditions) in the public sector. This situation was generated because of the inadequacy of resources available for public care and resulted in the creation of the “AUGE Voucher” in early 2011, by which those insured by FONASA could also be seen by private providers should the time limit guarantee not be respected. This has permitted healthcare centers to decongest, balance weight within the system (not just the public sector) and also, rapidly reduce waiting lists. In September 2011 this had helped reduce the number of cases to just over 16,000. This “AUGE Voucher” is one of the first formal steps taken by the public sector to try to integrate the public and private providers, meaning that the public subsidy given to FONASA beneficiaries could be used in the private sector.

Far less clear is the effect that the reform has had on medical attention of diseases not included in AUGE. However, the existence of waiting lists for AUGE treatments and strong political pressure on health service managers to reduce these was a strong indicator that in practice, AUGE had shifted resources and attention from other diseases. The existing information on non-AUGE waiting lists is practically non-existent. According to a private study done on a small set of non-AUGE diseases in October 2007, the number of cases of these pathologies on waiting lists was twice that corresponding to AUGE diseases. Additionally, 83% of the non-AUGE waiting lists were for as long as two months or more [15]. A similar result was found by Gonzalez, which was based on the study of an AUGE disease (Chronic Renal Insufficiency) during the testing phase of the reform [16]. Recently, it was informed that there are non-vital interventions (like tonsil operations, hernias, and hip surgeries) that have an average wait of 3.5 years. On a national level, there are more than 69 thousand surgeries on hold [17]. By 2011, according to FONASA there are more than 89 thousand cases on the waiting list [18]. Waiting lists in both AUGE and non-AUGE conditions are indicative of the reform being implemented with capacity constraints in both medical infrastructure and human resources. For instance, the density of physicians in Chile is 10.9 per 10000 population, whereas in comparable Argentina, Brazil and Uruguay such figure is 31.6, 17.2 and 37.4, respectively. The density of nurses and midwives in Chile is 6.3 per 10000 inhabitants, while it is 4.8, 65 and 55.5 in Argentina, Brazil and Uruguay, respectively. The number of hospital beds per 10000 inhabitants in Chile is 21, while it is 41 in Argentina, 24 in Brazil and 29 in Uruguay [19].

There are no impact assessments on the AUGE reform, although some studies have reported partial indicators of some of its effects. Infante and Paraje identified, for example, the groups that have contributed to financing the reform (basically made from public funds and from an increase in the VAT tax rate) [4]. It has been argued that even though the taxes used to finance the reform are regressive, it is the low-income group that receives a higher net subsidy for health benefits linked to AUGE. Additionally, there has been a strong increase of medical tests performed in the public health system, resulting from the investment in diagnostic equipment (like scanners, for example). Bitrán et al. showed that six chronic conditions included in AUGE (arterial hypertension, diabetes mellitus type I and II, depression, childhood epilepsy and HIV/AIDS) have had significant changes in health outcomes [5]. The proportion of cases that receive treatment (calculated over a potential demand) has increased noticeably and the mortality rates of these conditions have declined (in some cases like depression or childhood epilepsy have had a 99% decline). Hospitalization rates have decreased for some conditions (hypertension, diabetes mellitus type I and HIV/AIDS), as a consequence of improved outpatient treatments, while others have increased (Type II diabetes mellitus, childhood epilepsy, depression) as a consequence of greater coverage.

Nazzal et al. found that hospital mortality for acute myocardial infarction declined significantly after the implementation of AUGE and attributes this to the adoption of treatments based on medical evidence (from the definition of treatment protocols) [20]. Concha et al. found significant differences pre- and post-AUGE in the prenatal detection of congenital heart disease (possibly due to the inclusion of prenatal tests in the AUGE) [21]. Both studies are based on observations in a group of hospitals (the former ten hospitals, the latter two).

Infante and Paraje showed that the financial protection given by AUGE to the families of the lower decile (at least in its first stage of implementation) is moderate for two reasons [4]. First, because AUGE did not represent anything new in this area for families belonging to FONASA groups A and B (with free care in the public system before the implementation of AUGE); and secondly, because the main health expense for these families was the purchase of medicine; this item was not only not diminished with the AUGE reform but has increased.

The evaluation of AUGE by users and the general population has been diverse over time. From its implementation until 2010, the positive considerations towards the benefits granted by AUGE increased for FONASA and ISAPRES beneficiaries alike [22]. Table 4 is based on data from CASEN 2009, and it shows that the level of user satisfaction in terms of time and healthcare quality compliance is similar among beneficiaries of both types of insurers. The greatest satisfaction is obtained, however, among members of the ISAPRES system.

**Table 4:** Perceived time and quality compliance in AUGE healthcare conditions

**Perceived time compliance**						
	**FONASA A + B**	**FONASA C + D**	**ISAPRE**	**None**	**Another/Don’t know**	**Total**
Very good	20.8	20.8	29.9	18.1	19.3	21.1
Good	50.7	48.7	50.5	41.3	51.2	50.4
Fair	18.0	17.5	13.9	22.7	19.2	17.9
Bad	6.1	7.9	2.3	8.4	5.4	6.2
Very bad	2.7	3.2	1.0	2.5	2.3	2.7
Don’t know	1.6	1.9	2.4	6.9	2.5	1.8
Total	100.0	100.0	100.0	100.0	100.0	100.0
**Perceived quality compliance**						
	**FONASA A + B**	**FONASA C + D**	**ISAPRE**	**None**	**Another/Don’t know**	**Total**
Very good	21.3	22.4	28.3	17.2	18.6	21.6
Good	52.1	49.1	53.8	39.7	51.3	51.6
Fair	16.9	16.2	11.4	24.6	21.7	16.9
Bad	5.3	6.7	3.1	8.7	3.7	5.4
Very bad	2.7	3.2	0.8	1.2	1.9	2.6
Don’t know	1.7	2.4	2.5	8.6	2.8	2.0
Total	100.0	100.0	100.0	100.0	100.0	100.0

Since 2010, these positive evaluations have diminished, but are still higher than the negative ones. The main issues that influenced the negative evaluations are related to existing waiting lists, inability to choose providers and quality of care received (especially for the beneficiaries of FONASA). Also, since the beginning of the reform there has been a significant lack of awareness about how to use the guarantees, which even include doubts about what AUGE is and what it consists of (although this lack of knowledge has tended to decline over time). It is expected that the reduction in waiting lists will improve the opinion of AUGE. The evolution of user perception of the health system (not just respecting AUGE) in general is less clear, and has been adversely affected by waiting times (also in non-AUGE diseases) and the general quality of care received.

### Methodology and data source

#### Concentration index (CI) and horizontal inequity index (HI)

In order to analyze the effect on inequality and horizontal inequity in the use of medical services pre and post-AUGE reform, we used a decomposition of concentration and horizontal inequity indices, proposed by Zhong [6]. This decomposition allowed us to consider whether changes in these indices (pre and post-reform) were produced by an increase in the widespread use of medical services (“mean effect”) or if they were the result of a change in the socioeconomic distribution in the utilization of these services (“distribution effect”).

The concentration index has been widely used in literature on health inequalities because of its easy calculation and interpretation (analogous to the well-known Gini coefficient). Basically, the index ranks individuals or households according to some socioeconomic stratification variable (income, expenditure, assets, education, etc.) and compares such a ranking with the distribution of a health outcome or healthcare use variable [23]. The mathematical expression that defines the concentration index is:

(1) $$C=\frac{2\mathrm{cov}\left(y,R\right)}{\mu }$$

where cov (*y,R*) is the covariance between the health outcome (with a population mean equal to *μ*) and the relative rank of individuals in terms of a socioeconomic variable (income, for example). In the event that the socioeconomic distribution of the health variable is “pro-rich” (i.e. it is relatively concentrated among individuals of higher socioeconomic status) the concentration index is positive and is negative otherwise. The closer it is to 1 (in absolute value), the higher the socioeconomic concentration of the health variable.

On the other hand, horizontal inequity in healthcare use measures how differently health services are used among individuals with equal needs, according to their socioeconomic level. Wagstaff and van Doorslaer propose a method considering the necessity of medical attention (from the definition of variables measuring these needs, such as sex, age, etc.), by excluding factors not associated with them, like socioeconomic variables, for example [24]. Thus, for each individual the “needed” use of medical consultations is estimated, using information about their particular variables of need and the sample mean of variables unrelated to need. The estimate for each individual can be interpreted as the number of medical consultations that they would receive, given their personal characteristics, if treated like the rest who have the same characteristics. So if the health outcome (number of medical consultations, for example) is explained by the regression:

(2) $$h_{i}=\alpha +\sum_{j}\beta_{j}x_{\mathrm{ji}}+\sum_{k}\gamma_{k}z_{\mathrm{ki}}+\varepsilon_{i}$$

where *h*_*i*_ is the health outcome/use for individual *i*, *x*_*ji*_ is a vector of “need” variables (i.e. variables proxying for medical needs such as age, sex, disability, etc.), *z*_*ki*_ is a set of “non-need” variables (income, education, occupation, etc.), *α*, *β*_*j*_ and *γ*_*k*_ are parameters to be estimated. These parameters are used along with the mean of the “non-need” or socioeconomic variables to estimate the expected value of the health variable (or “necessary” use of services, in this case):

(3) $$h_{i}^{+}=\hat{\alpha }+\sum_{j}{\hat{\beta }}_{j}x_{\mathrm{ji}}+\sum_{k}{\hat{\gamma }}_{k}{\overset{―}{z}}_{k}$$

The estimation of use of medical attention by (indirect) standardization is equal to [25]:

(4) $$h_{i}^{\mathrm{IS}}=h_{i}-h_{i}^{+}+h^{\mu }$$

where *h*^*μ*^ corresponds to the sample mean of the respective health variable. From this, it is possible to calculate the horizontal inequity index (HI) as the concentration index of the variable *h*^*IS*^. A positive (negative) value of the HI indicates pro-rich (poor) inequity.

Assuming that *h*^*max*^ denotes the health variable of interest for the year that has the highest mean value and *h*^*min*^ the same variable for the year in which the mean is the lowest, thus, for each individual *i* it is possible to decompose the variable

(5) $$h_{i}^{\max}=h_{i}^{\min}+d_{i}$$

If the concentration index of *h*^*max*^ is *C* and for *h*^*min*^ is C*, it can be shown that [16]:

(6) $$C=\left(1-d\right)C^{*}\;+\;dC^{D}$$

where *d* is the ratio between the mean of *d*_*i*_ and *h*_*i*_^*max*^; and *C*^*D*^ is the concentration index of *d*_*i*_. In this case:

(7) $$\mathrm{\Delta C}=C-C^{*}=d\left(C^{D}-C^{*}\right)$$

The parameter *d* can be interpreted as the rate of change in the health variable’s average and as such, shows a “mean effect”. The rest of the equation measures the “distribution effect” of change in the concentration indices. If the distribution of *d*_*i*_ is more pro-rich than *h*^*min*^ (*C*^*D*^ > C^*^), the change in inequality is positive (the distribution of the health variable becomes more pro-rich). Otherwise, it becomes more pro-poor. In the case of *C*^*D*^ = C^*^, there are no changes in inequality over time even if there is an increase (decrease) in the use of health services. Finally, if there is an equal increase in the use of these services for all individuals (*C*^*D*^ =0), the change in inequality is equal to –dC^*^.^d^

From the horizontal inequity index it is also possible to determine what portion of change from one period to another is due to changes in the average use of medical care (*h*^*μ*^) and how much is due to changes in the socioeconomic pattern of deviations between what is used and what is considered necessary (*h*_*i*_ - *h*_*i*_^*+*^). Zhong showed that [6]:

(8) $$\begin{array}{l} \mathrm{\Delta HI}=\mathrm{HI}-{\mathrm{HI}}^{*}\\ \;=\left(\frac{1}{1-d}\mathrm{HI}-{\mathrm{HI}}^{*}\right)-\left(\frac{d}{1-d}\mathrm{HI}\right) \end{array}$$

The first term in parentheses measures the change in horizontal inequity keeping the average use constant (“distribution effect”), while the second term in parentheses reflects what happens to horizontal inequity when only the average use of health services changes (“mean effect”).

### Econometric models

Following Grossman, it is assumed that health is produced within each household and that the use of healthcare can be explained by individual-level and household-level variables that determine such use [26]. A reduced-form relationship is thus estimated. This relationship does not intend to explain potential demand (there is no information on prices, for example) nor access (no information on supply factors), but healthcare utilization.

Considering the fact that healthcare use is a discrete variable the most appropriate model to estimate the equation is a count data model. There is a wide range of count models; some of them consider the possibility of a high concentration of non-users, that is individuals who have not sought any medical care in the period preceding the survey. For instance, the Zero-Inflated Poisson (ZIP) or Generalized Negative Binomial (GNB) provide a relatively high weighting to the probability that the count variable (visits or use of medical care) is zero [27,28]. Using any of them involves considering the use of healthcare services as a two-decision process. The first decision models participation, like the decision of whether or not to use the healthcare service (a binary decision); while the second decision considers the intensity of this use (given the positive response to the first decision, the second is a count variable). Models like the GNB or ZIP integrate both decisions into the same estimation (contrary to, for instance, “hurdle” models that separate them).

We estimated several econometric models for the different utilization variables used to estimate HI, including linear models (OLS), Poisson, negative binomial, Zero-inflated Poisson (ZIP), IP, Zero-inflated negative binomial (ZINB), Generalized Negative Binomial (GNB) and standard “hurdle” models. The OLS model was discarded for theoretical reasons: it is continuous and allows negative predictions [27]; from the other models only one was selected: the one which presented the best values of the Akaike Information Criterion and the Bayesian Information Criterion (as used in [29,30]). Following both criteria, the best model was the generalized negative binomial (GNB), whose results are reported here (in the Additional file 1: Appendix Table A.1 and Additional file 2: Appendix Table A.2). The *gnbreg* command in STATA 12 was used to estimate the model. Estimation was done considering the sampling strategy of the surveys (through the *svy* set of commands).

### Data

The data comes from the Socioeconomic Characterization surveys (CASEN) collected by the Ministry of Planning. These surveys are representative at the national, regional and community levels and cover both urban and rural areas. They possess information on income and socioeconomic characteristics of households and their members, as well as information about the use and usage intensity of medical services. Specifically, there are questions about the number of individuals’ visits to general practitioners, specialists and emergency rooms in the three months preceding the survey. There is also information on the number of laboratory tests, x-rays and ultrasounds performed in the same period and number of hospitalization days in the year preceding the survey. In this paper we used the CASEN 2003 (pre-reform) and CASEN 2009 (post-reform). The former has 257,077 observations and the latter 246,925.

However, the information contained in both surveys on medical care is not fully comparable. For specialist care, CASEN 2009 separates the mental healthcare from the rest of the specialties. These were combined to ensure comparability with 2003. Thus, the dependent variables in the estimated models are the number of visits to general practitioners; specialists; emergency room visits; the number of laboratory tests; X-rays and ultrasounds; and hospitalization days.

Gender and age were considered within the individual explanatory variables. For women, the age groups defined were 0–4 years, 5–14 years, 15–34, 35–44, 45–59 and 60 and over. For men, the segments were 0–4 years, 5–14 years, 15–44, 45–64 and 65 and older. In both cases, biological factors were taken into account (beginning childbearing age, menopause, retirement, etc.) for the definition of these segments. Additionally, we considered the existence of a physical disability of some kind and if the person belonged to an aboriginal ethnic group.

Regarding the educational level of individuals, three categories were used: low education (completed basic education or less), middle education (completed secondary education) and higher education (tertiary or university studies, complete or incomplete). If the individual was below 21 years old and attending school or university, her value for this variable was her mother’s or the household head’s education (in that order). If the individual was older than 21, or younger but not attending school, her schooling level according to the aforementioned categorization gave her value for this variable. This criterion was chosen to be able to include individuals younger than 15 (usually not considered in this type of analysis). For these cases, the mother’s education is considered following evidence on its effect on child health status [31,32].

Working status was considered as an individual-level variable (with two categories: employed and unemployed or inactive) unless the person was younger than 15 years. In this case, the head of the household determined the value for this variable. Marital status was considered in two possible categories: married or live-in partner and others. In the case of children under 18 years old the value of this variable was determined by the mother or by the head of household (in that order), unless the minor was already married or cohabiting.

Finally, household-level variables taken into account were: area, political region, income per equivalent adult, and type of health insurance. For the latter, three categories were considered: FONASA (any of the four funds), ISAPRES (which includes the beneficiaries of the armed forces and other private insurance) and no insurance at all. The area was a dichotomous variable (urban/rural) and the political region was one of the 13 regions that existed in 2003 or one of the 15 regions in 2009.

Both surveys contained information on cash income for each household member from labor and capital sources, as well as monetary subsidies granted by the State. They also contained information on non-cash payments and the imputed rental value of home ownership. However, only cash income was considered here to determine total income per equivalent adult, which is total household income divided by the number of “equivalent” members and adjusted by a household economy-of-scale parameter [33]:

(9) $$\begin{array}{l} \mathrm{No}\;\mathrm{Equivalent}\;\mathrm{Adults}=\left(\mathrm{No}\_14\_\mathrm{older}\right)\\ \;{\left(+0.75*\mathrm{No}\_\mathrm{less}\_14\right)}^{0.75} \end{array}$$

where *No_14_older* is the number of individuals 14 and older 14 in the household, *No_less_14* is the number of children younger than 14. The power parameter is the household economy-of-scale, measuring the existence of “intra-household public goods”. Total individual income per equivalent adult (IEA) is, thus:

(10) $$\mathrm{IEA}=\frac{\mathrm{Total}\;\mathrm{Household}\;\mathrm{Income}}{\mathrm{No}\;\mathrm{Equivalent}\;\mathrm{Adults}}$$

Table 5 shows the descriptive statistics of total household income per equivalent adult and the use of healthcare interventions by geographic area for CASEN 2003 and 2009.

**Table 5:** Descriptive statistics of medical care use

	**2003**			**2009**			**Percentage change 2003-2009**		
**Number of visits**	**Urban**	**Rural**	**Total**	**Urban**	**Rural**	**Total**	**Urban**	**Rural**	**Total**
General practitioner	1,940,990	283,960	2,224,950	3,822,906	443,012	4,265,918	97.0	56.0	91.7
Emergency room visits	1,294,417	144,879	1,439,295	1,512,464	175,868	1,688,332	16.8	21.4	17.3
Specialist Doctors	2,929,237	240,908	3,170,145	3,427,421	225,962	3,653,383	17.0	−6.2	15.2
Laboratory Exams	1,876,600	182,586	2,059,186	3,739,192	412,163	4,151,355	99.3	125.7	101.6
X-ray and ultrasound	853,060	59,911	912,971	1,300,733	129,433	1,430,166	52.5	116.0	56.6
Hospitalization days	6,910,579	1,058,445	7,969,024	6,526,848	1,192,165	7,719,013	−5.6	12.6	−3.1
**Mean**									
Income	218,693	110,721	204,560	322,079	190,821	305,300	47.3	72.3	49.2
General practitioner	0.1443	0.1402	0.1437	0.2648	0.2100	0.2578	83.5	49.9	79.4
Emergency room visits	0.0962	0.0715	0.0930	0.1046	0.0833	0.1019	8.7	16.5	9.6
Specialist Doctors	0.2177	0.1189	0.2048	0.2374	0.1071	0.2207	9.0	−9.9	7.8
Laboratory Exams	0.1395	0.0901	0.1330	0.2594	0.1957	0.2513	86.0	117.2	88.9
X-ray and ultrasound	0.0634	0.0296	0.0590	0.0903	0.0613	0.0866	42.4	107.4	46.8
Hospitalization days	0.5137	0.5224	0.5148	0.4496	0.5634	0.4642	−12.5	7.8	−9.8
**Standard Deviation**									
Income	121.6	177.7	108.6	136.7	198.2	122.4	-	-	-
General practitioner	0.0002	0.0004	0.0002	0.0003	0.0005	0.0002	-	-	-
Emergency room visits	0.0001	0.0003	0.0001	0.0002	0.0003	0.0001	-	-	-
Specialist Doctors	0.0003	0.0006	0.0002	0.0003	0.0005	0.0003	-	-	-
Laboratory Exams	0.0002	0.0004	0.0002	0.0003	0.0006	0.0003	-	-	-
X-ray and ultrasound	0.0001	0.0002	0.0001	0.0001	0.0003	0.0001	-	-	-
Hospitalization days	0.0012	0.0031	0.0001	0.0011	0.0034	0.0010	-	-	-

Chile 2003 and 2009.

Source: Own calculation based on CASEN.

Table 5 shows that the average total individual income per equivalent adult in the urban sector has been consistently higher than rural and that such income at current prices increased almost 50% between 2003 and 2009. Regarding healthcare use, mean visits to general practitioners rose sharply (79% at the national level) as did laboratory tests (89% across the board). In addition, the mean of emergency room visits grew (10%), as did consultations with specialists (8%) and X-rays and ultrasounds (47%). On the other hand, the length of hospital stays decreased (10%).

While the increase in use of healthcare services may not be directly associated to the reform (from the point of view of the data emerging from the CASEN there is no visible difference between AUGE and non-AUGE consultations) there is no doubt that the reform has increased the overall use of health services (based on data emerging from other sources such as FONASA or the Superintendence of Health). Therefore, whether the increased medical attention was AUGE-related or not, it is legitimate to analyze this change (and its socioeconomic pattern) as a result of the reform. This does not imply that all change in the use of healthcare services are due strictly to the reform, as there may be changes in other relevant variables (such as family income, relative prices, preferences for health care, etc.). In the absence of information on these variables, this paper assumes that these variables (or at least their distribution) remain unchanged between 2003 and 2009.

## Results

The concentration indices for general practitioners care, specialists, emergency room, laboratory tests, X-rays and ultrasounds and hospital days were estimated from CASEN 2003 and 2009 data. Additionally, we estimated the Gini coefficient of total individual income per equivalent adult. These data are shown in Table 6.

**Table 6:** Concentration indices (CI)

**Concentration Index**	**2003**	**2009**
Income	0.5446	0.5157
General practitioner	−0.0265	0.0275
Emergency room visits	−0.1034	−0.0978
Specialist Doctors	0.1835	0.1583
Laboratory Exams	0.1362	0.1084
X-ray and ultrasound	0.1809	0.1175
Hospitalization days	−0.0710	−0.0785

Source: Own calculation based on CASEN.

The income concentration index (Gini coefficient) shows a slight decline between 2003 and 2009. However, it remains at a high level showing a highly concentrated distribution of income (Chile has one of the world's highest Gini). The specialized care, lab tests and X-rays and ultrasounds had strong pro-rich distributions. However, in all cases this decreased between 2003 and 2009. Visits to general practitioners went from a moderately pro-poor CI to moderately pro-rich. By contrast, emergency care and inpatient days have concentration indices showing a pro-poor distribution, which is relatively stable over time. This inequality pattern (pro-poor for general practitioners and pro-rich for specialists) has been frequently observed in literature from both developed and developing countries [6,34,35].

Table 7 shows the decomposition of the concentration index for medical care, while Table 8 displays the decomposition of horizontal inequity indices for different types of medical attention. Both tables show mean utilization of medical care services, concentration indices (both for 2003 and 2009) and changes in those variables. In addition, they show the estimates of the mean and distribution effects for those changes and, in the last column, which of them prevails (if any).

**Table 7:** Decomposition of concentration indices

	**Mean**		**Concentration index**			**Change**	**Decomposition**		
	**2003**	**2009**	**2003**		**2009**	**CI**	**Mean effect**	**Distribution effect**	**Result**
General practitioner	0.1437	0.2589	−0.0265	(*)	0.0275	0.0540	0.4450	0.1214	Mean effect
Emergency room visits	0.0929	0.1025	−0.1034		−0.0978	0.0056	0.0933	0.0600	Mean effect
Specialist Doctors	0.2048	0.2217	0.1835		0.1583	−0.0253	0.0764	−0.3307	Distribution effect
Laboratory Exams	0.1330	0.2520	0.1362		0.1084	−0.0278	0.4722	−0.0589	Mean effect
X-ray and ultrasound	0.0589	0.0868	0.1809		0.1175	−0.0635	0.3214	−0.1974	Mean effect
Hospitalization days	0.5148	0.4685	−0.0710		−0.0785	0.0075	0.0899	0.0834	Non-defined

All Cis are statistically significant at 99%, except (*) that is significant at 95%.

**Table 8:** Decomposition of Horizontal inequity (HI) Indices

	**Mean**		**Horizontal inequity index**			**Change**	**Decomposition**		
	**2003**	**2009**	**2003**		**2009**	**HI**	**Mean effect**	**Distribution effect**	**Result**
General practitioner	0.1437	0.2589	−0.0273	(*)	0.0465	0.0738	0.0372	0.1110	Distribution effect
Emergency room visits	0.0929	0.1025	−0.0968		−0.0737	0.0231	−0.0076	0.0155	Distribution effect
Specialist Doctors	0.2048	0.2217	0.1784		0.1824	0.0041	0.0151	0.0191	Non-defined
Laboratory Exams	0.1330	0.2520	0.1128		0.1170	0.0042	0.1047	0.1089	Non-defined
X-ray and ultrasound	0.0589	0.0868	0.1703		0.1317	−0.0386	0.0624	0.0238	Mean effect
Hospitalization days	0.5148	0.4685	−0.0789		−0.0564	−0.0225	−0.0078	−0.0303	Distribution effect

All Cis are statistically significant at 99%, except (*) that is significant at 95%.

## Discussion

The fact that some of these healthcare services have a pro-poor distribution (as shown in Table 6) does not necessarily show a desirable situation. The greater relative use of emergency care and hospitalization by the poor may come from the fact that when they do access the system it is in more critical situations than average. There is no information that directly corroborates this hypothesis, but indirect evidence is given from the type of payment made in each intervention. During 2003, for example, of those who attended a general practitioner, 58% had free medical care (outside the ISAPRES). In the case of specialists that percentage was 37% and in the case of emergency treatment was 64%. In 2009 the percentage of individuals who received free care from general practitioners and specialists was 60% and 42% respectively. In the case of emergency treatment, the percentage was 70%. This would imply that for both years (pre and post-AUGE) a significantly higher percentage of those who used emergency room visits did not pay for their visits, as compared to other medical visits. This could indicate that in the case of general or specialized care, relatively poor individuals choose to postpone or avoid a visit since in a high percentage of cases they have associated costs. In the case of emergency care there is a lower possibility of doing the latter.

In terms of changes of inequality (Table 7), with the exception of specialists’ care and hospitalization days, what has dominated the change in the concentration index pre and post-reform is the mean effect. In the case of general practitioners, average use increased strongly between 2003 and 2009 and, in turn, the rate went from slightly pro-poor to slightly pro-rich. This implies that the “extra care” that occurred in this period was carried out on individuals with a relatively high socioeconomic ranking. The opposite happened with specialized care, the average use of specialized doctors rose moderately in this period, but their distribution became more pro-poor (or rather, less pro-rich since it was still mostly concentrated on individuals of higher socioeconomic status). In fact, if we estimate the concentration index of the greatest number of healthcare consultations between 2003 and 2009, it has a value of −0.15, which is strongly pro-poor. While it is not possible to link these results directly to the reform (because they do not control other variables such as price changes, income, new services, etc.) there is a striking contrast between these two types of medical services. If it is believed that conditions included in the AUGE reform are intensive in specialized care (beyond the first stage of diagnosis), it is a legitimate hypothesis that the distribution effect observed in specialists is a consequence of the reform.

In the case of laboratory tests, X-rays and ultrasound scans, the mean effect prevailed but there were differences between them. For laboratory tests, the increase in average use had a distribution reflecting a pro-rich socioeconomic pattern (although less than the original), while the pattern was slightly pro-poor in the case of X-rays and ultrasound. Again, there is no direct evidence that this has been linked to AUGE, although the increase in imaging equipment investments (in scanners, for example) suggests indirect evidence that the mean effect found in this area is a result of the reform.

Finally, in the case of hospitalization days, the effect is undefined because the mean effect has practically the same magnitude as the distribution effect. In this case, the average fell between 2003 and 2009, but the concentration index remained the same. The reduction of inpatient days followed a pro-poor pattern, which means that the poor reduced their hospitalization days more than the rich. This could be in line with better healthcare in stages before hospitalization (making it unnecessary to reach hospitalization) or more advanced surgical techniques (and previously unavailable) requiring shorter hospital stays.

When horizontal inequity is taken into consideration (Table 8) the panorama changes significantly. First, it should be mentioned that during 2009 all HI, with the exception of X-rays and ultrasounds became more pro-rich or less pro-poor, compared to 2003. The use of general practitioners moved from a pro-poor HI to a slightly pro-rich one, which indicates that once needs are considered, relatively rich people increased their use of this service between 2003 and 2009 beyond their (expected) needs. In fact, this is what was observed by decomposing. The distribution effect was higher than the mean effect, which implies that the near doubling of visits to general practitioners was mainly explained by variables unrelated to need and related to socioeconomic factors.

The emergency procedures showed similar results. In both 2003 and 2009 the HI was pro-poor which shows us that after considering needs, it was the relatively poor people that made more use of this service. The average use of the same service rose slightly in 2009 over 2003, but became less pro-poor. In this case, however, and given that the average use increased slightly, the distribution effect is only marginally higher than the mean effect.

In the case of medical specialists, in both 2003 and 2009 the HI was strongly pro-rich, a signal that after considering needs it was high-income people that made more use of this service. Between these years the average use increased moderately (which would decrease the HI) but the HI became more pro-rich. Both tended to be opposite effects, without one dominating over the other.

The same is true for laboratory tests, which rose sharply between 2003 and 2009, whereas HI remained constant. This would indicate that the sharp increase in the use of this service was primarily intended to cover the common needs for all socioeconomic groups. In the case of X-rays and ultrasound scans, the sharp increase in the use of these implies a decrease in HI between 2003 and 2009 (the extra benefits were concentrated among lower socioeconomic groups). In this case, the mean effect prevailed over the distribution effect.

Finally, in the case of hospitalization days the average usage decreased and the HI became less pro-poor. The distribution effect prevailed slightly over the mean effect, meaning that the higher socioeconomic groups were less affected by the decrease in the use of this service after considering their specific needs.

## Conclusions

The results of the previous section, although preliminary in the absence of systematic data on the impact of the AUGE reform on equity and being only a comparative statics exercise, show some clear conclusions. The first is that between 2003 and 2009, the average use of medical care increased, and in some cases quite significantly. The increase in the average number of visits to general practitioners, laboratory tests, X-rays and ultrasounds showed a very significant mobilization of resources for medical care. This is consistent with the increase in spending per beneficiary of public health insurance and the large equipment purchase in the public healthcare system. Many of the conditions covered in AUGE require research and analysis for detection and monitoring, which was the main reason why this investment in equipment was made.

The second conclusion is that in terms of inequity the increase in average use has not always led to an improvement in its socioeconomic distribution. In fact, the horizontal inequity index (HI) increased in many cases. Although this increase was modest for medical specialists and laboratory tests, it was important for other services such as general practitioners and emergency care. Both types of healthcare services, whose use is more prevalent among lower income groups, decreased their pro-poor profile in this period. This result is curious in a period where a reform that held equity improvement as one of its main pillars was implemented. According to estimates provided herein, equity in the use of the majority of healthcare services decreased. The exceptions are the use of x-rays and ultrasounds and hospitalization days.

The fact that there was an increase in horizontal inequity during this period does not invalidate the reform but casts doubt on whether it has been the best strategy to achieve the equity goal. The reform sought to improve equity by increasing access to certain treatments for socioeconomic groups that were relatively neglected before the reform. However, the reform has provided universal access to these treatments. There has been no specific strategy for focusing such increased access and, as such, it has benefited individuals across the socioeconomic gradient. This may be linked to the concept of reform itself: the AUGE reform sought to reach the poorest individuals through universal benefits. This strategy, associated with the rights-based approach in health and the necessity for political approval of the reform, would result in a dissipation of the benefits from this reform mainly among higher socioeconomic groups.

In addition, there is evidence that the implementation of the reform occurred under capacity constraints. Human resources in health (and also infrastructure) were already relatively small before the reform. Increased access to guaranteed conditions seems to have aggravated this constraint to the point of generating waiting lists in both AUGE and non-AUGE conditions. Evidently, waiting lists are severely biased towards low socioeconomic patients. Increasing the number of conditions guaranteed by AUGE (inclusion that not always followed equity criteria) may have aggravated this problem.

A natural question that arises from these results is whether a reform like AUGE was needed to increase the use of healthcare services. In recent years, even before the reform, the amount of resources destined to FONASA was significant, resulting in partial closure in the health expenditure gap with ISAPRES. The consequences of the reform, in terms of healthcare use, aimed to strengthen the previous trend and channel it towards a certain group of conditions resulting in a reallocation of resources within the public sector. Results show that prioritization has occurred, instead of an across-conditions increase. This has not yet happened among ISAPRES beneficiaries, which may explain why, after the reform, equity in healthcare use has not increased.

## Endnotes

^a^The contribution by the Central Government to FONASA as a proportion of FONASA’s total expenditures went from 45% in 2002 to 60% in 2010. This relative increase occurred during a persistent period of growth due to increased membership and benefits received by FONASA beneficiaries.

^b^Type II diabetes mellitus, hypertension and acute respiratory infection were guaranteed from the beginning of AUGE, while depression and ambulatory dental emergencies were added later (the first in 2006 and the second in July, 2007).

^c^These are: terminal chronic renal insufficiency, diabetes mellitus type II, arterial hypertension, depression in people 15 years or older, dental emergencies, HIV/AIDS, preventive medical exam, and breast cancer in women 15 years or older.

^d^This result is consistent with what happens in the Gini Index: if income increases by the same amount across individuals, the index decreases.

## Competing interests

The authors declare that they have no competing interests.

## Authors’ contributions

GP has 1) made substantial contributions to conception and design, analysis and interpretation of data; 2) has been involved in drafting the manuscript; and 3) have given final approval of the version to be published. FV has 1) made substantial contributions to conception and design, analysis and interpretation of data; 2) has been involved in drafting the manuscript; and 3) have given final approval of the version to be published.

## Supplementary Material

Additional file 1 **Appendix Table A.1.** Generalized Negative Binomial Regression Coefficients. Chile 2003. Click here for file

Additional file 2 **Appendix Table A.2.** Generalized Negative Binomial Regression Coefficients. Chile 2009. Click here for file
